# An analysis of gene expression profiles through machine learning uncovers the new diagnostic signature for diabetic foot ulcers

**DOI:** 10.3389/fgene.2025.1620749

**Published:** 2025-06-24

**Authors:** Yingnan Li, Ning Xiao, Zhuoqun Wang, Wenhai Wang, Fengjiao Li, Jiren Wang

**Affiliations:** ^1^ Hand and Foot Surgery and Burn and Plastic Surgery, Jilin Province FAW General Hospital, Changchun, Jilin, China; ^2^ Office of Clinical Trial Institutions, Jilin Province FAW General Hospital, Changchun, Jilin, China; ^3^ Department of Neurology, Jilin Province FAW General Hospital, Changchun, Jilin, China; ^4^ Department of Cardiology, Jilin Province FAW General Hospital, Changchun, Jilin, China; ^5^ Department of Anesthesiology, Jilin Province FAW General Hospital, Changchun, Jilin, China

**Keywords:** diabetic foot ulcers, WGCNA, PPI network, melanin synthesis, diagnosis

## Abstract

**Purpose:**

Diabetic foot ulcers (DFUs), a serious diabetes complication, greatly increase disability and mortality, underscoring the need for effective diagnostic markers.

**Methods:**

We used GSE199939 and GSE134431 datasets from the Gene Expression Omnibus (GEO) database, removed batch effects, and identified differentially expressed genes (DEGs). The weighted gene co-expression network analysis (WGCNA) was used to identify co-expression modules, followed by the integration of the protein-protein interaction (PPI) network to screen key genes, which were further optimized using LASSO regression. The gene set enrichment analysis (GSEA) analyzed key gene-related pathways, CIBERSORT assessed immune infiltration, and potential target drugs were predicted using the DGIdb database.

**Results:**

We identified 403 DEGs in DFUs, intersected them with 2,342 genes from a DFU-related WGCNA module to find 193 overlapping genes, and screened candidates via PPI network. LASSO regression finalized *DCT*, *PMEL*, and *KIT* as the key genes. GSEA analysis showed these three genes may influence the MAPK and PI3K-Akt pathways and were positively correlated with Dendritic. cells.resting. Drug target prediction identified 85 potential drugs for *KIT*, six for *DCT*, and six for *PMEL*.

**Conclusion:**

This research highlights *DCT*, *PMEL*, and *KIT* as diagnostic biomarkers for DFUs, which are linked to melanin production and the MAPK/PI3K-Akt signaling pathways.

## Introduction

Diabetic foot ulcers (DFUs) represent a significant complication associated with diabetes mellitus, characterized by persistent nonhealing wounds resulting from diabetic neuropathy affecting sensory, motor, and autonomic functions, along with vascular issues and bacterial infections (Armstrong et al., 2023). About 34% of people with diabetes are likely to experience a foot ulcer at some point in their lives, and those with DFUs have a mortality rate that is 2.5 times greater over 5 years compared to diabetics without foot ulcers (Armstrong et al., 2017; Rehman et al., 2023). DFUs are managed conservatively and often present early (Al-Rubeaan et al., 2015). When experiencing resting pain, surgical intervention is often required. Techniques for restoring blood circulation to the lower limbs involve arterial bypass, the placement of stents, along with various surgical and interventional methods (Bandyk, 2018). Utilizing spinal cord stimulation (SCS) may aid in relaxing the blood vessels in the lower limbs, enhancing the microcirculation in the extremities, and to some extent, preventing ulcers and gangrene, while also promoting healing and repair, which could reduce the likelihood of amputation (Li et al., 2021). Nonetheless, regardless of whether surgical intervention or SCS therapy is employed, complete reconstruction of blood vessels or treatment solely for vascular conditions is not achievable, and there is a considerable risk of long-term recurrence (Islamov et al., 2022). Consequently, there is an urgent need to identify and develop diagnostic and therapeutic biomarkers for DFUs with high specificity.

Biomarkers, described as “attributes of objective assessment and measurement that serve as indicators of typical biological functions, disease processes, or pharmacological reactions to treatment,” play a crucial role in the early diagnosis of clinical conditions, prevention of diseases, and forecasting the progression of illnesses (Wang et al., 2021). In recent years, numerous genes associated with DFUs have been identified as potential biomarkers for disease diagnosis. For example, Jeandrot et al. identified C-reactive protein as a reliable biomarker for distinguishing different DFUs grades in a study involving DFUs patients and diabetic controls (Jeandrot et al., 2008). Weigelt and colleagues indicated that the levels of IL-6 in individuals suffering from acute DFUs were elevated compared to those in diabetic patients who did not have diabetic foot issues (Weigelt et al., 2009). Additionally, Mir and his team discovered a correlation between the HSPA1B genotype, which pertains to the polymorphism of heat-shock protein (HSP) 70, and both the severity of DFUs as well as the results of surgical interventions (Mir et al., 2009). Despite the identification of numerous individual biomarkers through various methods, there is still a deficiency in model analyses that focus on multiple key genes in DFUs. Therefore, transcriptomics-based to explore novel biomarkers for the treatment and diagnosis of DFUs is imperative.

Melanin synthesis plays a crucial role in maintaining skin pigmentation. Additionally, melanin and its metabolites help maintain skin microenvironment homeostasis by scavenging reactive oxygen species, reducing oxidative damage, and modulating the release of inflammatory factors (Solano, 2020; Fu et al., 2022). While melanin is traditionally associated with skin coloration, recent studies have hinted at its role in antioxidant defense, cellular homeostasis, and stress response modulation (Braydon et al., 2024). Recent studies have shown that abnormalities in melanin synthesis may impair the proliferation and differentiation of skin cells, thereby disrupting the normal wound healing process (Sato et al., 2024). In this study, we aim to employ transcriptome analysis to uncover the possible critical molecular mechanisms involved in the development of DFUs. By integrating differential expression analysis, co-expression network construction, and functional enrichment, we aim to identify a previously unexamined class of biomarkers, potentially uncovering novel biological pathways contributing to DFU development. Furthermore, immune infiltration analysis was conducted to explore the relationship between key genes and the DFUs microenvironment. Finally, drug prediction was performed to identify potential therapeutic targets. These findings provide novel insights into the molecular landscape of DFUs, offering promising biomarkers for early diagnosis and potential therapeutic strategies for clinical intervention.

## Methods

### Data collection and integration

We obtained the dataset from the Gene Expression Omnibus (GEO) database (https://www.ncbi.nlm.nih.gov/geo/). The GSE199939 dataset was obtained from the Illumina NovaSeq 6,000 (*Homo sapiens*) sequencing platform, which includes 10 samples of DFUs and 11 control samples. The GSE134431 dataset was obtained from the Illumina NextSeq 500 (*H. sapiens*) sequencing platform, which includes 13 DFUs samples and eight control samples. Next, we used the “sva” package to merge the two datasets after removing the batch effect, and the resulting merged dataset contained a total of 23 samples of DFUs and 19 normal control samples.

### Screening and enrichment analysis of differentially expressed genes (DEGs)

Differential expression analysis was performed using the ‘limma’ package in R. DEGs were selected using false discovery rate and significance analyses, applying the criteria of |log2FC|>1.5 and adjust. P value <0.05. Heatmaps were created to illustrate the findings. Subsequently, the clusterProfiler package in R was utilized to perform analyses on DEGs between normal samples and DFUs, focusing on Gene Ontology (GO) terms—encompassing biological processes, cellular components, and molecular functions—as well as Kyoto Encyclopedia of Genes and Genomes (KEGG) pathways. GO and KEGG terms with a significance level of *p* < 0.05 were identified as significantly enriched.

### Weighted gene co-expression network analysis (WGCNA)

The “WGCNA” package in R was utilized as described in earlier studies (Peter and Steve, 2008). In summary, we performed hierarchical clustering on genes according to their expression values for the targeted genes, grouping those with similar high expression into the same module using the dynamic tree cut method. Subsequently, we computed the Module Eigengene (ME) for each module and assessed the correlation coefficient between the ME and the specific phenotype of interest. A P value of less than 0.05 was established as the threshold for determining significance in the correlation analysis between ME and phenotype. A higher absolute value of ME indicates a stronger correlation between the module and the phenotype of concern.

### Protein‐protein interaction (PPI) network analysis

STRING (https://string-db.org/) is widely regarded as a comprehensive database designed for the analysis and prediction of protein interactions and functional relationships related to human diseases (Szklarczyk et al., 2019). In our study, we employed STRING to examine the interactions of proteins and their functional relationships through the analysis of DEGs and WGCNA. Subsequently, we utilized Cytoscape software (https://cytoscape.org/index.html) to create a visual representation of the PPI network. Meanwhile, the entire network was performed using the MCODE plugin in Cytoscape software and clusters were constructed using default parameters for further screening of genes.

### Construction of LASSO model and validation of key genes

The LASSO model can perform variable screening while fitting a generalized linear model. For this purpose, we used the glmnet package of R language in order to perform the construction of LASSO model (Friedman et al., 2010), which in turn further screened the key genes associated with DFUs. Specifically, 10-fold cross-validation for LASSO regression was employed, and each dataset was randomly divided into ten subsets of similar sizes. In each iteration, one subset functioned as the validation set, while the other nine subsets were merged to create the training set. This procedure was repeated ten times, with each subset serving as the validation set once. During cross-validation, the mean squared error (MSE) was calculated for each model configuration. The optimal regularization parameter (*λ*) for LASSO regression was determined by selecting the value that minimized the average MSE across all ten folds, which effectively mitigates overfitting and enhances the model’s generalizability. Subsequently, the ROC package in the R software was applied to evaluate the sensitivity and specificity of the screened key genes by receiver operating characteristic (ROC) curves and the area under curve (AUC) values.

### Gene set enrichment analysis (GSEA)

DFUs samples from the merged dataset were used for GSEA. We arranged the DFUs samples in descending order according to each therapeutic target gene and defined samples above the median gene as high expression groups and samples below the median expression as low expression groups to perform GSEA between groups. Pathways that were significantly enriched were finally screened for *p* value < 0.05.

### Immune infiltration analysis

The software known as CIBERSORT will analyze the composition of immune infiltrating cells by utilizing a gene expression matrix along with a predefined set of 547 barcode genes, employing an inverse convolution algorithm. The combined proportions of all predicted immune cell types in every sample will total 1. In this context, the CIBERSORT algorithm is applied to determine the relative proportions of 22 immune cell types within the DFUs samples. Wilcoxon rank sum test was utilized to assess the differences in immune cell infiltration between DFUs and control subjects. Subsequently, the correlation coefficients and p-values relating to key genes and each immune cell were computed via the Pearson method.

### Drug‐gene interaction

The DGIdb database (https://dgidb.genome.wust-l.edu) is used to predict drug-gene interactions (Cotto et al., 2018), which in turn provide potential treatments for DFUs. Key genes with the screened genes were entered into the database and an interaction score >1 was selected to map the drug network. The resulting drugs should include those that have received approval and demonstrate a well-defined pharmacological effect, whether as inhibitors or activators.

### Statistical analysis

In this study, all statistical analyses were performed by R software (version 4.3.2). The “pROC” package was used to produce ROC curves to demonstrate whether the target gene has diagnostic value. In addition, Pearson correlation analysis was performed using the R language “cor” function. Differences were considered statistically significant at *p* < 0.05.

## Results

### Screening and pathway enrichment analysis of DEGs in DFU

**FIGURE 1:**
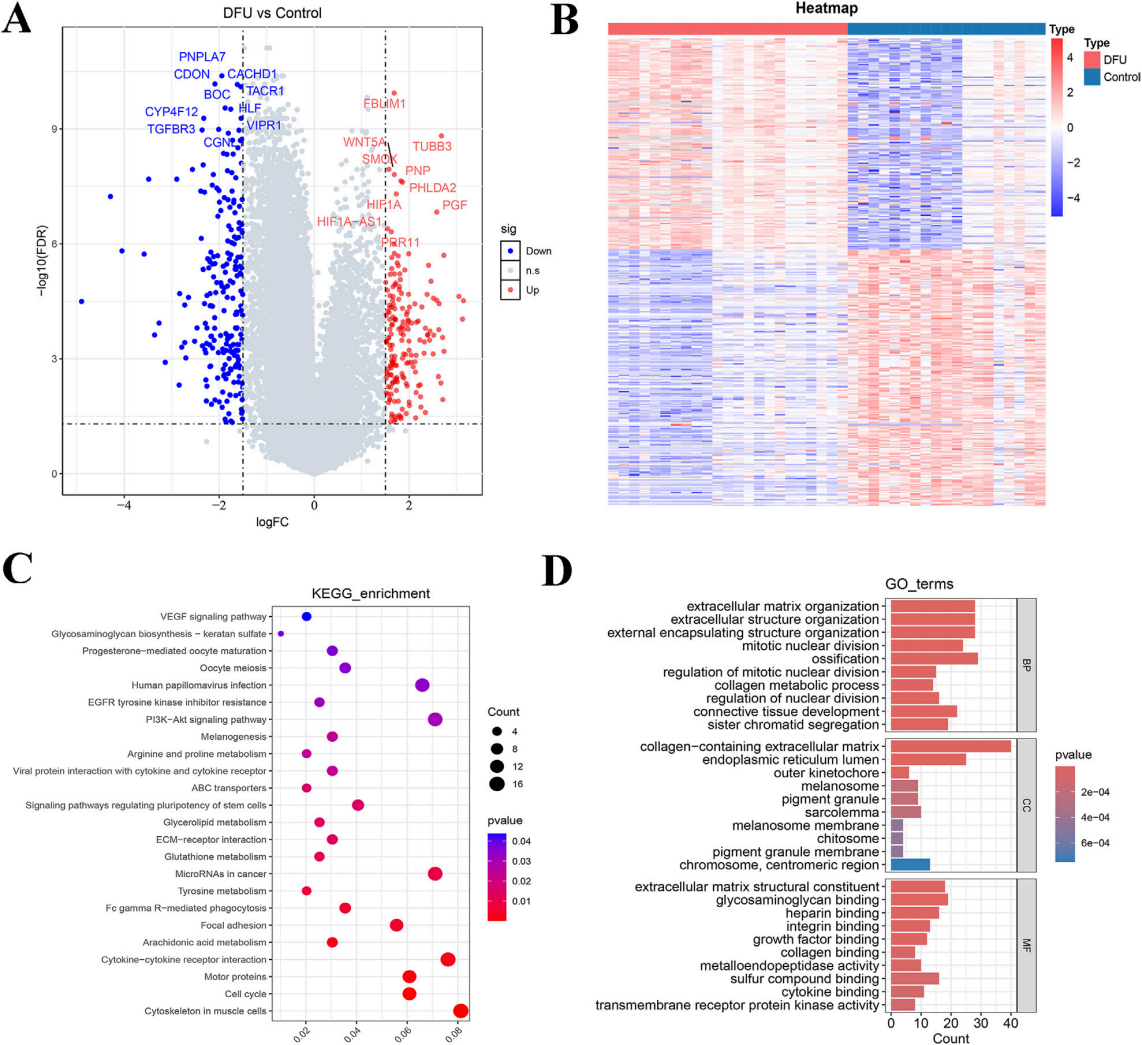
Based on GSE199939 and GSE134431 datasets to screen differentially expressed genes for DFUs and enrichment analysis. **(A)** Volcano plot showing the distribution of differentially expressed genes between DFUs and controls. **(B)** Heat map of differentially expressed genes. **(C)** KEGG pathway enrichment analysis of differentially expressed genes. **(D)** GO function enrichment analysis of differentially expressed genes. BP, biological process; CC, cellular component; MF, molecular function.

Firstly, 23 samples of DFUs and 19 control samples reached by the combined dataset (GSE199939 and GSE134431). Subsequently, 403 DEGs (including 182 upregulated genes and 221 downregulated genes) were obtained by performing differential analysis (Figures 1A,B). The enrichment analysis showed that 403 DEGs were significantly enriched for a total of 24 KEGG pathways, such as cytoskeleton in muscle cells, cell cycle, and motor proteins (Figure 1C; Supplementary Table S1). These DEGs exhibited significant enrichment across 1,103 biological process (BP) terms, highlighting pathways such as extracellular matrix organization, extracellular structure organization, and regulation of nuclear division pathway. In the cellular component (CC) category, 62 terms were enriched, including melanosome, pigment granule, and melanosome membrane pathways. Additionally, 85 molecular function (MF) terms were significantly associated with these DEGs, particularly extracellular matrix structural constituent, sulfur compound binding, and cytokine binding (Figure 1D; Supplementary Table S1).

### Identification of critical gene modules in DFUs

**FIGURE 2:**
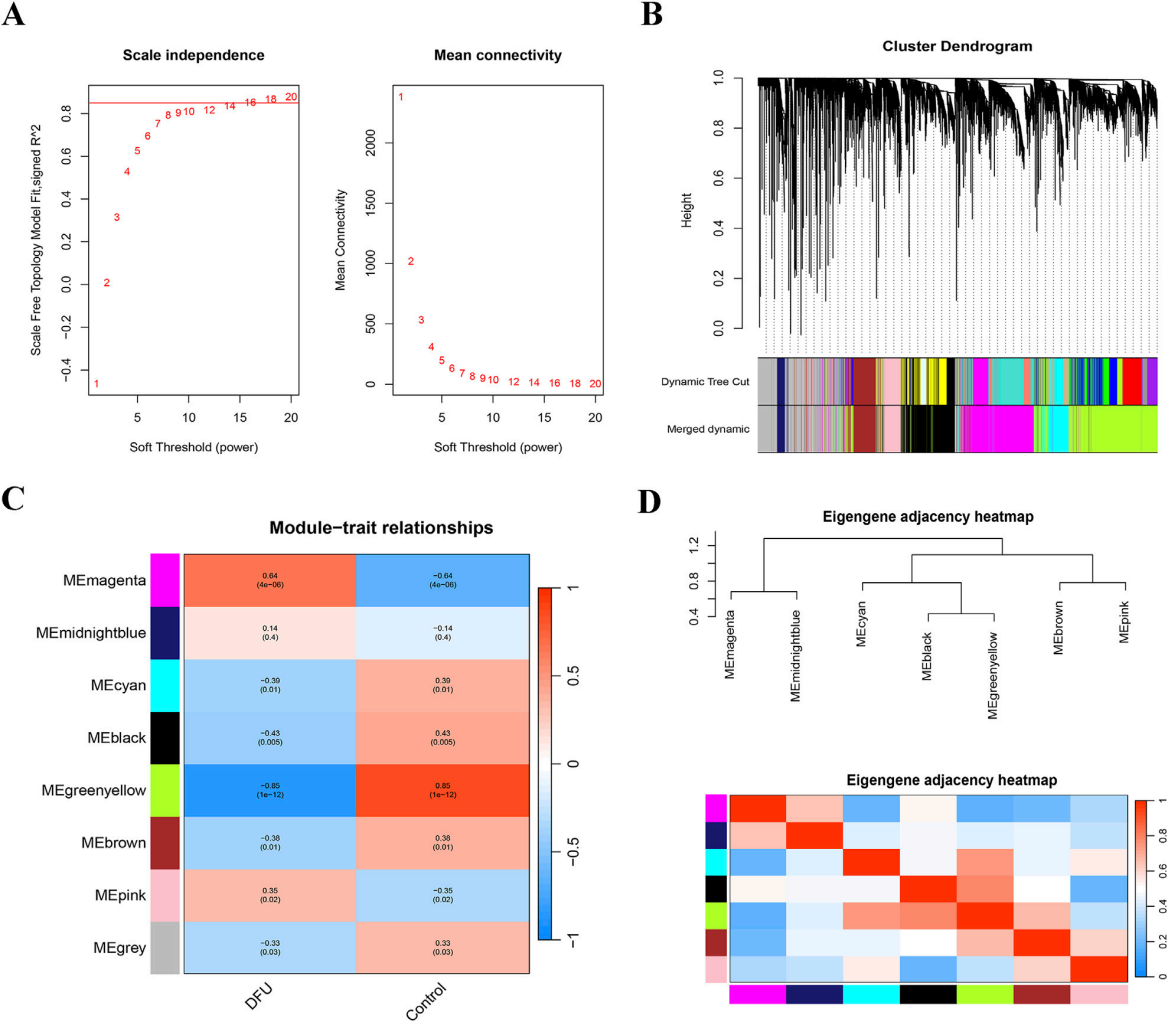
WGCNA was constructed based on the GSE199939 and GSE134431 datasets. **(A)** Histogram representing the distribution of connectivity at β = 16 (left), and an examination of the scale-free topology at β = 16 (right). **(B)** Cluster dendrogram of gene co-expression modules, each color represents a gene module. **(C)** Heatmap of correlation between DFUs samples and module characterization genes. Red represents positive correlation, blue represents negative correlation, darker color means stronger correlation, and the number in each cell indicates correlation and significance. **(D)** Eigengene adjacency heatmap.

In this study, we employed WGCNA to identify essential modules related to DFUs. The integrated datasets (GSE199939 and GSE134431) were classified into eight distinct gene modules, using the topological overlap matrix (TOM) and an optimal soft threshold of β = 16 (Figures 2A,B). Next, we categorized the samples in the cohort into DFUs and Control samples and made them as trait data for WGCNA. We calculated the correlation between modules and sample traits using the Pearson method and identified the MEgreenyellow module as the key module most associated with DFUs, containing a total of 2,342 genes (Figure 2C; Supplementary Table S2). Furthermore, the MEgreenyellow module also demonstrates significant independence compared to the other screened gene modules, suggesting that it could be crucial in the progression of DFUs (Figure 2D).

### Identification and analysis of key genes in DFUs

**FIGURE 3:**
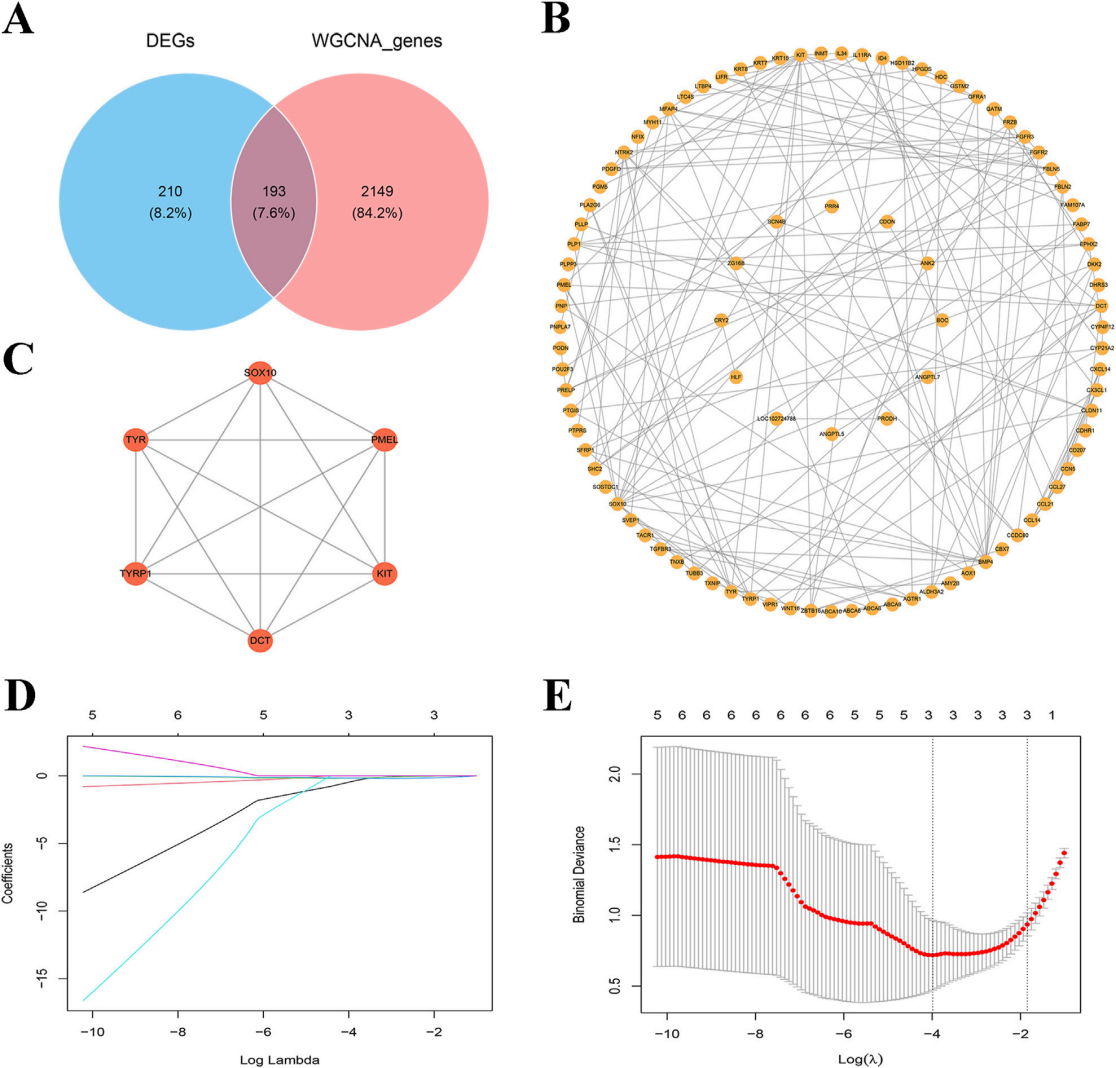
Screening of key genes for DFUs. **(A)** Venn diagram of differentially expressed genes (DEGs) versus WGCNA genes. **(B)** Co-expression network map of key genes. Each node represents a gene and edges represent interrelationships between genes. **(C)** PPI networks for key genes. **(D)** Path diagram of regression coefficients for the LASSO regression model. **(E)** Cross-validation curves for LASSO regression. λ = 3 as the optimal parameter.

To further screen for biomarkers associated with DFUs, we took the intersection of 2,342 MEgreenyellow module genes and 403 DEGs and obtained a total of 193 intersecting genes (Figure 3A). Subsequently, to investigate the relationships among these essential genes, we conducted a PPI network analysis with the help of the STRING database. This interaction network consisted of 95 nodes and 156 edges, which were visualized through the Cytoscape software (Figure 3B). We utilized the MCODE plugin to detect gene cluster modules. Based on the filtering criteria, we identified seven cluster modules, with the highest scoring module (score: 6.000, including 6 nodes and 15 edges) (Figure 3C). Therefore, these six genes (including *DCT*, *SOX10*, *PMEL*, *KIT*, *TYR*, and *TYRP1*) were identified as candidate genes associated with DFUs at the protein interaction level. In the LASSO model, a λ value of 3 was selected as the best variable selection criterion, which led to the identification of genes *DCT*, *PMEL*, and *KIT* as key genes for DFUs (Figures Figure3D–E).

### Identifying of diagnostic markers in key genes associated with DFUs

**FIGURE 4:**
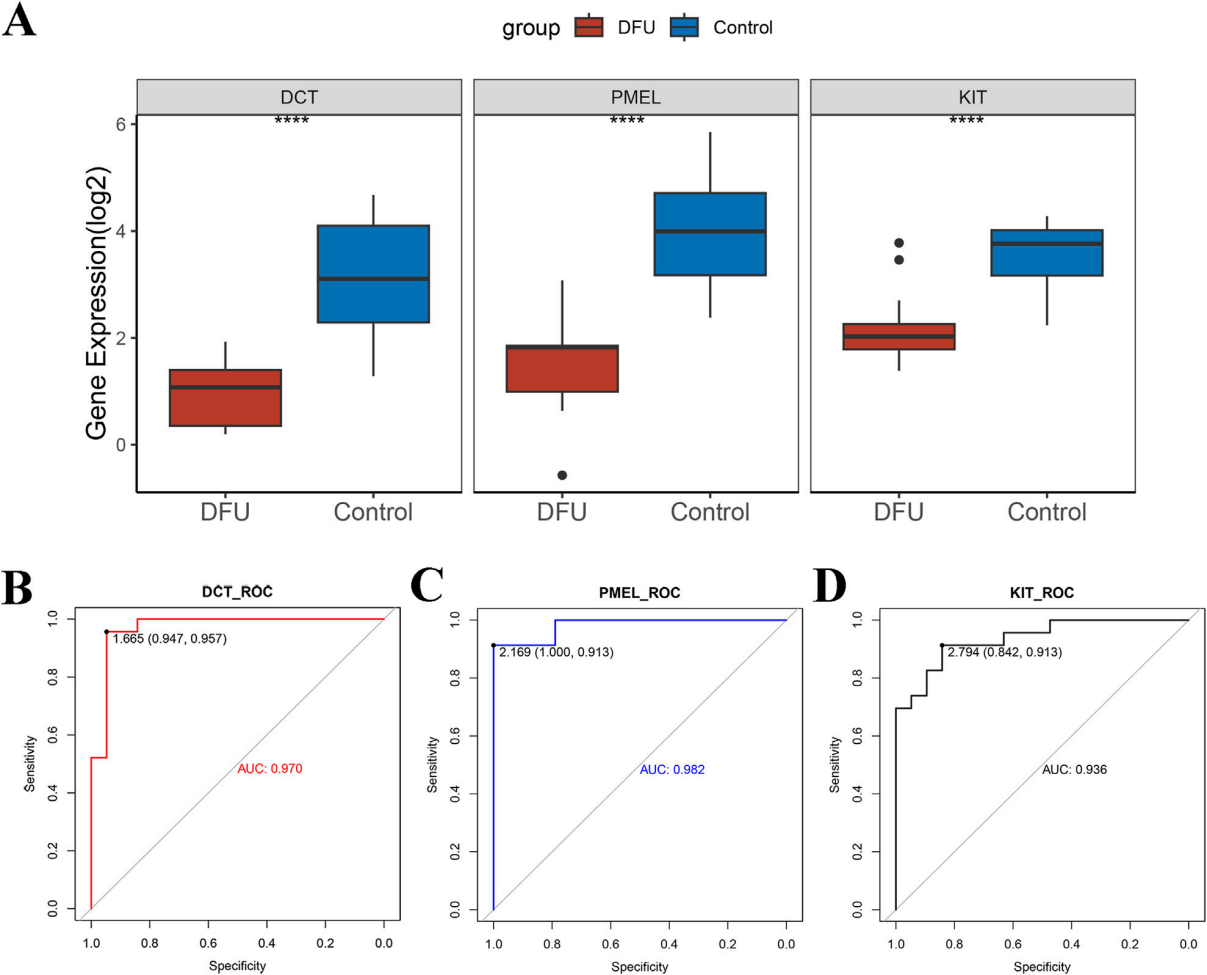
Validation of potential therapeutic targets for DFUs. **(A)** A combined dataset based on GSE199939 and GSE134431 was used to validate the expression differences of *DCT*, *PMEL*, and *KIT* between DFUs samples and control samples. **(B–D)** ROC curves to demonstrate the diagnostic capabilities of the *DCT* **(B)**, *PMEL* **(C)**, and *KIT* **(D)**. **** stands for *p* < 0.0001.

To enhance the accuracy of predicting DFUs progression, we initially employed the Wilcoxon rank sum test to examine the expression differences of these three key genes (*DCT*, *PMEL*, and *KIT*) between DFUs samples and control groups. Our analysis revealed that these genes were notably downregulated in DFUs (Figure 4A). Subsequently, we generated ROC curves to assess the diagnostic effectiveness of *DCT*, *PMEL*, and *KIT*. As illustrated in (Figures 4B–D), the AUC values for these three genes exceeded 0.9, demonstrating their strong predictive capability. Specifically, the AUC for *DCT* was 0.970, for *PMEL* it was 0.982, and for *KIT* it was 0.936, all of which indicate significant diagnostic potential (AUC >0.9). These results imply that these 3 key genes we screened may serve as potential therapeutic targets for DFUs.

### Investigation of unique signaling mechanisms associated with signature genes in DFUs

**FIGURE 5:**
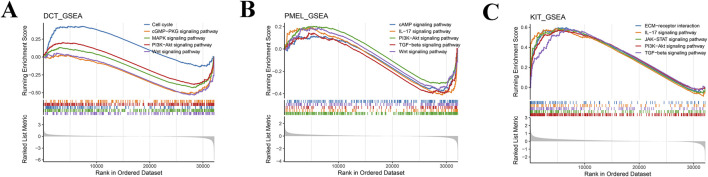
GSEA of key genes. GSEA enrichment analysis pathway maps for *DCT* **(A)**, *PMEL* **(B)**, and *KIT* **(C)**. A line graph of gene Enrichment Score with the horizontal axis for each gene under the gene set and the vertical axis for the corresponding Running ES. The lower part of the graph shows the distribution of rank values for all genes.

Subsequently, GSEA analysis showed that a total of 90 pathways were significantly enriched in the high *DCT* expression group relative to the low *DCT* expression group, such as Cell cycle, cGMP-PKG, MAPK, PI3K-Akt, and Wnt signaling pathway (Figure 5A; Supplementary Table S3). A total of 48 pathways were significantly enriched in the high-expression group of *PMEL* relative to the low-expression group, such as cAMP, IL-17, PI3K-Akt, TGF-beta, and Wnt signaling pathway (Figure 5B). Differential genes between the *KIT* high and low expression groups were mainly enriched in 153 pathways. Similarly, increased expression of *KIT* was associated with enrichment of the ECM-receptor interaction, IL-17, JAK-STAT, PI3K-Akt, and TGF-beta signaling pathway (Figure 5C). These results suggest that *DCT*, *PMEL*, and *KIT* may regulate DFUs progression through multiple signaling pathways, particularly MAPK, PI3K-Akt, and immune-related pathways.

### Immune profile of DFUs

**FIGURE 6:**
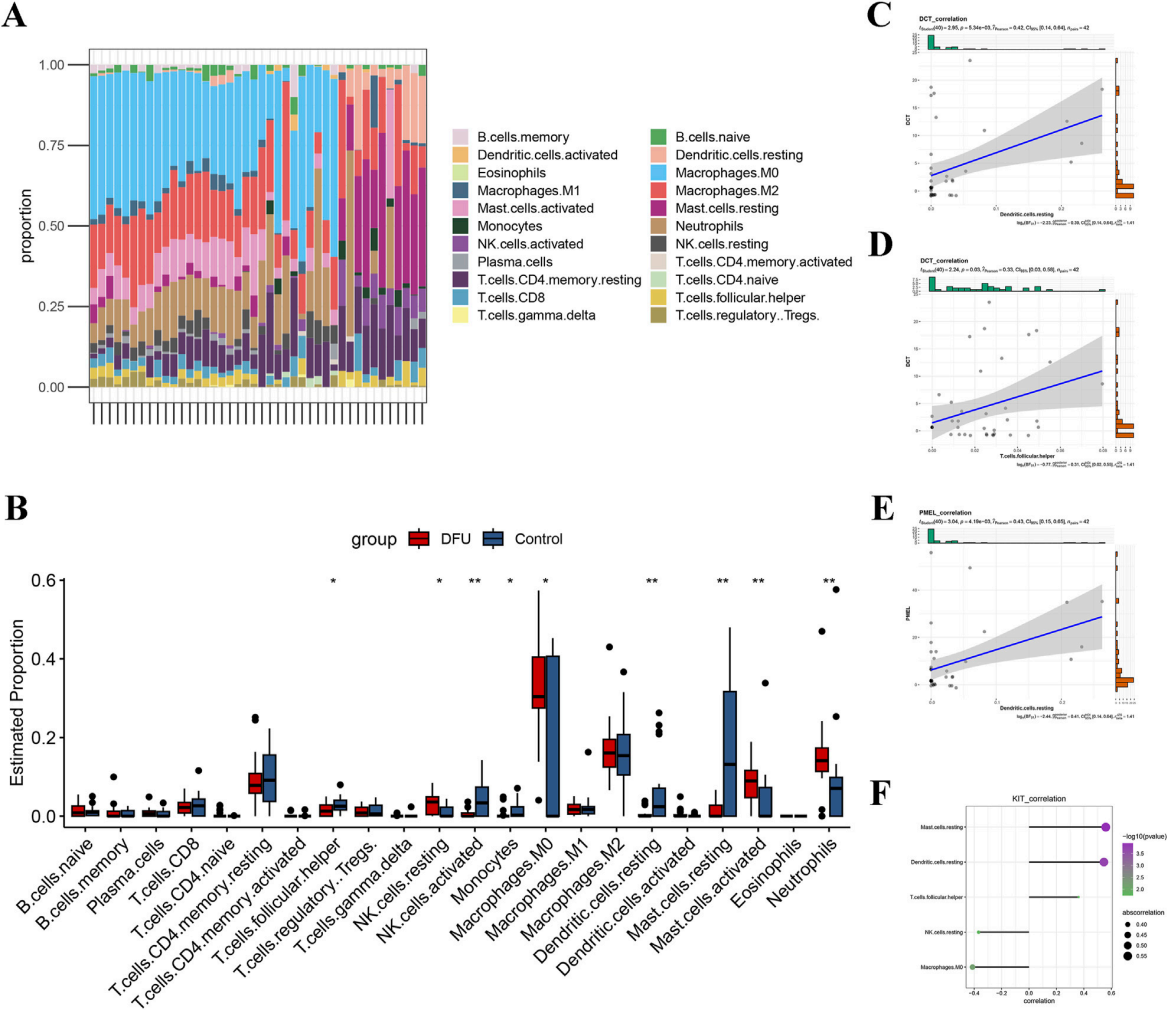
Assessment of immune cell infiltration in DFUs. **(A)** Bar-stack plot of the percentage of immune cell infiltration in DFUs. **(B)** Based on the CIBERSORT algorithm to assess differences in 22 immune cells in DFUs samples and control samples. **(C,D)** Scatterplot of correlation between DCT and Dendritic. cells.resting **(C)**, T. cells.follicular.helper **(D)**. **(E)** Scatterplot of correlation between PMEL and Dendritic. cells.resting. **(F)** KIT and immune cell (Mast.cells.resting, Dendritic. cells.resting, T. cells.follicular.helper, NK. cells.resting, and Macrophages. M0) correlation demonstrated. * represents *p* < 0.05, ** represents *p* < 0.01.

In order to explore the immunological landscape of DFUs more thoroughly, the CIBERSORT algorithm was employed to evaluate the relative abundance of 22 immune infiltrating cell types in both DFU samples and control samples. As shown in (Figure 6A), DFUs samples contained a high percentage of macrophages, mast cells, T cells, and neutrophils. The infiltration levels of nine types of immune cells, such as T cells follicular helper, NK cells resting, and NK cells activated, were significantly different between the DFUs samples and control samples groups (Figure 6B). Subsequently, we found a significant positive correlation between *DCT* and Dendritic. cells.resting and T. cells.follicular.helper, respectively (Figures 6C,D). *PMEL* was significantly positively correlated with Dendritic. cells.resting (Figure 6E). *KIT* was significantly positively correlated with T. cells.follicular.helper, Dendritic. cells.resting, and Mast. cells.resting, while it was significantly negatively correlated with Macrophages. M0 and NK. cells.resting (Figure 6F). The results indicate that the signature genes significantly impact the immune microenvironment in DFUs, highlighting their critical role in the immune features related to the condition.

### Prediction of potential drug targets in DFUs

**FIGURE 7:**
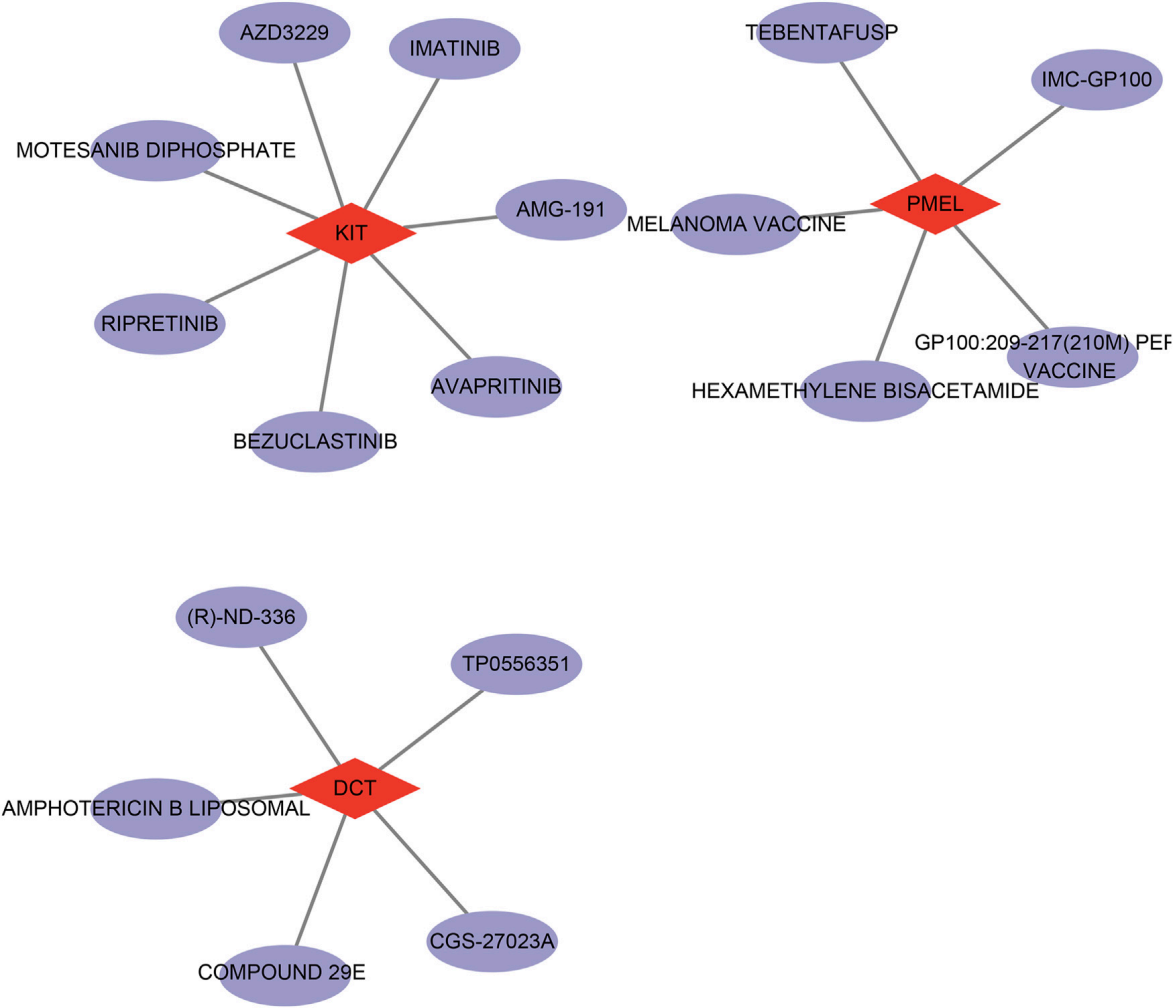
Network diagram of drug prediction results for therapeutic target genes (*DCT*, *PMEL*, and *KIT*).

Finally, we utilized the DGIdb database for drug target prediction for the 3 key genes and obtained a total of 6 drugs predicted by *DCT*, 6 drugs predicted by *PMEL*, and 85 drugs predicted by *KIT* (Supplementary Table S4). Next, we chose interaction score>1 to map the drug network and found that the possible target drugs for *KIT* include AZD3229, IMATINIB, AMG-191, etc. The possible target drugs for *PMEL* include MC-GP 100, MELANOMA VACCINE, etc. The possible target drugs for *DCT* include TP0556351, amphotericin B liposomal and CGS-27023A, among others (Figure 7). These findings indicate that *DCT*, *PMEL*, and *KIT* may serve as potential therapeutic targets for DFUs, and the identified drug candidates provide new possibilities for targeted treatment strategies aimed at improving wound healing and disease management.

## Discussions

Diabetic patients often experience DFUs, a serious complication that raises the likelihood of amputation and mortality due to their challenging healing process and high rates of recurrence (McDermott et al., 2023). In recent years, research has gradually revealed that DFUs are not only caused by local circulatory disorders and persistent inflammation but are also closely related to impaired skin barrier function and dysregulated antioxidant stress responses. These factors interact, leading to a state of chronic tissue damage in DFUs, further exacerbating the difficulty of wound healing (Armstrong et al., 2023). Therefore, it is imperative to identify the relative biomarkers with high specificity for the diagnosis and treatment of DFUs. In this study, we utilized the GEO database of DFUs to conduct differential gene and pathway enrichment analysis first. Consistent with previous studies, we found that the pathogenesis of DFUs involves vascular lesions, nerve damage, and infection (Fa-Shun et al., 2022; Huang et al., 2022). Specifically, based on the outcomes of GO enrichment pathway analysis, heparin binding was identified as significantly associated with vascular lesions, growth factor binding was demonstrated to exhibit a strong correlation with nerve damage. Likewise, integrin binding, cytokine binding, and transmembrane receptor protein kinase activity were identified to be linked to the infection process. These findings highlight the distinct functional implications of these molecular interactions in the context of specific pathological conditions. After that, based on the WGCNA and LASSO modeling, three target genes (*DCT*, *PMEL*, and *KIT*) related to melanin synthesis in DFU were identified.

It is noted that chronic hyperglycemia and persistent inflammation lead to increased oxidative stress in the pathological environment of DFUs, which may alter melanin production (Xu et al., 2022). Therefore, investigating the role of melanin synthesis in DFUs holds significant importance. Our findings indicate that these three key genes associated with melanin synthesis have the potential to be diagnostic and therapeutic targets for DFUs. Research indicates that the *DCT* phenotype is linked to increased neurotization in benign nevi and is also associated with ulceration in thin malignant melanomas (Filimon et al., 2014). *DCT* is primarily involved in melanogenesis and antioxidant defense, where it catalyzes the conversion of dopachrome to 5,6-dihydroxyindole-2-carboxylic acid (DHICA) in melanocytes and enhances melanin stability (Hearing, 2000). *PMEL* plays a crucial role as a structural protein in the development of melanosomes, facilitating the aggregation of melanin and the creation of fibrous structures to enhance both the storage and distribution of melanin (Zhang et al., 2021). Additionally, Pan et al. demonstrated that in HaCaT cells, a reduction in melanin correlates with a decline in the levels of *PMEL* protein, which is essential for the formation of melanosomes (Huang et al., 2024). *KIT* acts as a tyrosine kinase receptor and plays a central role in regulating melanocyte proliferation, survival and differentiation (Hu et al., 2020). Research indicates that melanin and its byproducts facilitate wound healing through the scavenging of reactive oxygen species, the attenuation of inflammatory factor release, and the promotion of skin barrier restoration (Qi et al., 2023). However, in patients with DFUs, we noted a significant decline in the expression levels of *DCT*, *PMEL*, and *KIT*. This reduction could result in diminished melanin production, consequently compromising the protective capabilities of the skin barrier, lowering antioxidant effectiveness, and increasing the wounds’ vulnerability to additional harm caused by oxidative stress and inflammation. Moreover, based on this discovery, we constructed ROC curves to evaluate the diagnostic efficacy of *DCT*, *PMEL*, and *KIT*. The results showed that the AUC values of these three genes all exceeded 0.9, suggesting their robust predictive power. This finding strongly indicates that *DCT*, *PMEL*, and *KIT* could potentially serve as therapeutic targets for DFUs in clinical practice.

The pathogenesis of DFUs is complex, involving the joint participation of multiple signaling pathways (Hu et al., 2024). In this study, GSEA analysis revealed that the three genes could affect the MAPK and PI3K-Akt signaling pathways, which provide a new insight for prospective exploration of the molecular mechanisms and therapeutic targets of DFUs. Aligning with our findings, previous studies in the pathological environment of DFUs, the abnormalities in these two signaling pathways may exacerbate impaired wound healing. In detail, the MAPK signaling pathway regulates the proliferation and migration of keratinocytes and fibroblasts during wound repair, and the elevated levels of MAPK are linked to the development in DFUs (Phimnuan et al., 2023; Shi et al., 2023). Likewise, the abnormalities of DFUs in the PI3K-Akt pathway may lead to reduced survival capacity of keratinocytes and endothelial cells, thereby impairing angiogenesis and wound closure (Xing et al., 2017). Functionally speaking, the signaling pathways MAPK and PI3K-Akt are pivotal in numerous biological processes, including cell growth, differentiation, survival, and the regulation of immune responses, which demonstrate significant activity across different physiological and pathological states, especially when it comes to the management of melanocyte activity, the production of melanin, and processes involved in skin repair (Corrales et al., 2022; Wu et al., 2024). The signaling pathways of MAPK and PI3K-Akt are crucial in regulating the synthesis of melanin. Research indicates that Selaginellin can impede melanogenesis by blocking the MAPK signaling pathway, which subsequently leads to a reduction in the expression of the microphthalmia-associated transcription factor (MITF) and the downstream genes tyrosinase (TYR) and tyrosinase-related protein 2 (TYRP2) (Zhou et al., 2022). In addition, as a G protein-coupled receptor, melanocortin 1 receptor (MC1R) manages the synthesis quantity and quality of melanin within melanocytes (Anyamanee and Uraiwan, 2022). During the process of melanin synthesis, the activation of MC1R was accompanied by an increase in cAMP levels and the subsequent MAPK signaling cascade, leads to the activation of MITF (Po-Yuan et al., 2018). Meanwhile, the signaling pathway involving PI3K-Akt also influences melanogenesis. Specifically, when this pathway is activated, it enhances the phosphorylation of MITF, boosts the survival of melanocytes, and modulates the expression of genes that are crucial for melanin production (Zhou et al., 2021). Moreover, PI3K-Akt is important for preserving the stability of melanocytes and providing resistance to oxidative damage, with its inhibition potentially resulting in compromised melanocyte function (Mosca et al., 2021).

Prior studies point out that NK cells play a significant role in the inflammation of DFUs, while the disease activity of DFUs is associated with macrophages (Wen-Juan et al., 2024). In line with that, the immune infiltration analysis in our study demonstrated that there were differences in the compositions of macrophages, mast cells, T cells, and NK cells. Specifically, the proportion of NK cells activated were significantly decreased in disease group, which further emphasizes the significance of immune response in the development of DFUs. Moreover, irregularities within the PI3K-Akt signaling pathway result in a reduction of M2-type anti-inflammatory macrophages (Deng et al., 2024). This shift consequently favors the dominance of M1-type pro-inflammatory macrophages, potentially sustaining an inflammatory condition in DFUs, ultimately impacting the healing process of the wounds (Jere et al., 2019). These findings suggest that the MAPK and PI3K-Akt signaling pathways may play key roles in the development of DFUs, and the aberrant expression of *DCT*, *PMEL* and *KIT* may regulate the DFUs microenvironment by affecting these signaling pathways, which offers a potential causal relationship between immune cells and DFUs. This provides an important theoretical basis for further exploration of the pathomechanisms of DFUs as well as targeted therapeutic strategies.

Identification of valid predictive diagnosis and drug targets are crucial for DFUs (Fang Leimi et al., 2022). In this study, the drug network was constructed, and the possible target drugs were predicted for DFUs. Imatinib, a Tyrosine kinase inhibitor, was found to be a target drug for *KIT*. Previous studies point out that imatinib could potentially influence related immunological and metabolic pathways, and preclinical investigations demonstrate that it can both reverse and prevent diabetes (Gitelman et al., 2021). Similarly, the melanoma vaccine indirectly regulates the synthesis of melanin by participating in immunomodulation, which was found to be a target drug for *PMEL* in the study (Yin et al., 2023). In addition, a study has shown that amphotericin B liposomal exhibits positive therapeutic effects on the complications of patients with diabetic foot (Muhamed et al., 2021). In line with this, our finding demonstrate amphotericin B liposomal was a potential target drugs for *DCT* in DFUs. Nonetheless, our current research has certain limitations. Firstly, the data utilized in this investigation were primarily sourced from the GEO database. Although we applied batch effect correction and cross-validation, there remains a potential bias, which stems from sample origin, processing differences, and population heterogeneity and could impact the generalizability of our findings. Additionally, despite our findings predicting potential drug targets for key genes in DFUs, our study did not undertake a preliminary evaluation of the applicability, safety, and efficacy of these targets in patient populations. Meanwhile, individual patient factors, such as age, diabetes duration, and comorbidities, were not incorporated to explore the therapeutic potential of identified targets. Subsequent research could benefit from the incorporation of independent datasets from multicenter clinical samples, encompassing data from patients with DFUs across various geographic locations and diverse populations, enhancing the generalizability and reliability of the findings. Secondly, the downregulation of *DCT*, *PMEL*, and *KIT* was noted in this study solely among a cohort of DFUs patients, leading to the hypothesis that these factors might influence the progression of DFUs through the MAPK and PI3K-Akt signaling pathways; however, direct causal evidence is absent.

In summary, our detailed analysis of the transcriptome emphasizes the crucial involvement of genes associated with melanin synthesis (*DCT*, *PMEL*, and *KIT*) in the advancement of DFUs and their notable relationship with the infiltration of immune cells. Additionally, our predictions regarding drug targets highlight possible therapeutic options. These results not only deepen our comprehension of the fundamental mechanisms underlying DFUs but also indicate that these biomarkers might act as novel therapeutic targets Further *in vitro* validation assays such as gene overexpression would be performed to observe the effects of changes in the expression of the three key genes (*DCT*, *PMEL*, and *KIT*) on cell proliferation, migration, apoptosis, and the pathological processes related to DFUs. Additionally, single-cell RNA sequencing should also be conducted for a more in-depth analysis of DFU heterogeneity, and animal models using CRISPR/Cas9 gene editing techniques would also be established to investigate their roles in the context of the disease. Lastly, we intend to leverage various drug screening databases in future investigations to discover compounds that may modify these genes and to carry out drug intervention experiments, such as treatments involving small molecule agonists or inhibitors in DFUs cellular models, to evaluate their impact on wound healing.

## Conclusion

In conclusion, the findings of this research indicate that there is an imbalance in the genes associated with melanin synthesis in DFUs. It offers new perspectives on the pathogenesis of DFUs and presents potential therapeutic targets for enhanced diagnosis and treatment.

## Data Availability

The original contributions presented in the study are included in the article/Supplementary Material, further inquiries can be directed to the corresponding author.
